# Dasatinib-Associated Chylothorax: A Scoping Review and Pharmacovigilance Analysis

**DOI:** 10.1007/s43441-026-00966-4

**Published:** 2026-05-11

**Authors:** Eleonora Castellana, Patricia Madalina Budau, Cecilia Miglietta, Paola Milla, Maria Rachele Chiappetta

**Affiliations:** 1 Hospital Pharmacy, University Hospital Città della Salute e della Scienza of Turin, Turin, Piedmont, Italy; 2 Università degli Studi di Torino, Turin, Italy; 3 School of Specialization in Hospital Pharmacy, University of Turin, Turin, Piedmont, Italy; 4 Department of Drug Science and Technology, University of Turin, Turin, Piedmont, Italy

**Keywords:** Dasatinib, Chylothorax, Leukemia, Myelogenous, Chronic, BCR-ABL positive, Drug-related side effects and adverse reactions, Pharmacovigilance, Spontaneous reporting systems

## Abstract

**Background:**

Dasatinib, a second-generation tyrosine kinase inhibitor widely used for chronic myeloid leukemia (CML) and Philadelphia chromosome-positive acute lymphoblastic leukemia (Ph + ALL), has been reported in association with rare cases of chylothorax.

**Objective:**

To systematically summarize published case reports and complement these findings with pharmacovigilance data in order to characterize the clinical presentation, management, and outcomes of dasatinib-associated chylothorax.

**Methods:**

A scoping review was conducted in accordance with PRISMA 2020 recommendations. PubMed/MEDLINE and an institutional discovery platform were searched for case reports and case series published up to February 13, 2026. Eligible reports included patients developing chylothorax during dasatinib therapy. In parallel, data from the FDA Adverse Event Reporting System (FAERS) were analyzed through September 30, 2024. Descriptive analyses were performed. FAERS data were interpreted as hypothesis-generating only.

**Results:**

Forty-two publications describing 44 patients met the inclusion criteria. Patients ranged from 5 to 90 years of age, with a slight male predominance. Time from dasatinib initiation to chylothorax onset varied from 2 months to 10 years. Management most commonly involved drug discontinuation and thoracentesis, frequently followed by clinical resolution. FAERS analysis identified 104 unique reports of chylothorax associated with dasatinib, all classified as serious adverse events; severe outcomes were uncommon. Reporting trends increased over time, particularly between 2017 and 2023.

**Limitations:**

Evidence derives from case reports and spontaneous reporting data, which do not allow estimation of incidence or causal inference and are subject to reporting bias and incomplete information.

**Conclusions:**

Dasatinib-associated chylothorax is an uncommon but clinically relevant event that may occur even after prolonged therapy. Long-term monitoring and prompt recognition are essential. Findings from pharmacovigilance data should be interpreted cautiously and considered hypothesis-generating. Further studies using large retrospective administrative databases are warranted to formally evaluate this potential association.

## Introduction

Dasatinib, a potent second-generation tyrosine kinase inhibitor, plays a crucial role in the treatment of chronic myeloid leukemia (CML) and acute lymphoblastic leukemia (ALL). Despite its high efficacy, its safety profile includes a range of adverse effects, among which chylothorax, though rare, is noteworthy. This complication, characterized by the leakage of chyle into the pleural space, represents a significant clinical challenge due to its potential severity and the lack of standardized therapeutic guidelines [1–3]. The pathogenesis of dasatinib-induced chylothorax remains an area of intense research and debate. Although growing clinical evidence suggests a possible relationship between dasatinib exposure and the occurrence of this complication, the precise mechanism is not yet fully understood [4, 5]. Several hypotheses have been proposed to explain this potential association. One of the most supported theories hypothesizes a direct effect of dasatinib on the thoracic duct or its tributary lymphatic vessels, which could contribute to chyle leakage into the pleural space [1]. This hypothesis is supported by observational data, as many reported cases occur within the first few years of treatment, although this temporal pattern alone is insufficient to confirm a direct toxic effect [2]. Another hypothesis posits that dasatinib may influence T lymphocyte function or inhibit the platelet-derived growth factor receptor (PDGFR), thereby disrupting lymphatic system balance and predisposing to chylothorax development [6]. However, this hypothesis requires further experimental validation [5]. It is also important to consider the potential influence of predisposing factors in the development of dasatinib-induced chylothorax. The presence of pre-existing conditions, such as cardiovascular diseases or hypertension, may increase susceptibility to this complication [7]. Moreover, the concomitant use of other drugs or treatments might interact with dasatinib, thereby raising the risk of chylothorax [8]. Finally, genetic or individual factors could play a role in the predisposition to this complication, although there is currently insufficient evidence to support this hypothesis. The duration of dasatinib treatment appears to be a factor associated with the onset of chylothorax; some studies suggest a higher incidence in patients treated for prolonged periods [2, 9]. However, cases of late onset, even after several years of treatment, have been reported, highlighting the complexity of this association [10].

The analysis of pleural fluid is essential for the diagnosis of chylothorax. The pleural fluid typically has a milky appearance, due to the presence of chyle, with a high triglyceride concentration (generally above 110 mg/dL) and a high lymphocyte count [2, 9]. However, it is important to emphasize that not all pleural effusions associated with dasatinib are chylous [11]. The presence of a pleural effusion with characteristics not suggestive of chylothorax requires careful evaluation to rule out other possible diagnoses through more detailed laboratory tests, such as the search for neoplastic cells or infectious agents [12]. Imaging techniques, such as chest radiography and computed tomography (CT), are useful for assessing the extent of the pleural effusion and guiding diagnostic and therapeutic procedures, such as thoracentesis [2, 13]. In some cases, lymphangiography may be necessary to identify the site of the lymphatic fistula and guide potential surgical intervention. The discontinuation of dasatinib is often the first therapeutic step, as stopping the drug may allow spontaneous resolution of chylothorax in some patients [2, 3, 14]. However, drug discontinuation alone is not always sufficient to achieve complete resolution, and in certain cases, chylothorax may persist even after treatment interruption [5]. In such cases, supportive measures are employed, such as low-fat dietary therapy, to reduce chyle production [2]. This diet, which limits the intake of saturated fats, can help decrease the amount of chyle produced and reduce the extent of the pleural effusion [2]. Diuretics may be used to reduce fluid accumulation, but their effectiveness in treating chylothorax is limited [2]. Corticosteroids, such as prednisone, may be administered to reduce inflammation and promote the healing of damaged lymphatic vessels [2, 4]. The prognosis of dasatinib-induced chylothorax is generally favorable, with most patients experiencing complete symptom resolution following drug discontinuation and the adoption of supportive measures [2, 3].

This work aims to conduct a comprehensive literature review and pharmacovigilance analysis regarding the reported association between dasatinib and chylothorax, with the objective of summarizing current evidence and generating hypotheses for future research rather than establishing causality.

## Materials and Methods

We performed a scoping review using the PRISMA methodology [15] and searched PubMed/Medline using the following search terms: ("dasatinib" OR "dasatinib") AND ("chylothorax" OR "chylothorax" OR "chylothoraxes"). A further search was conducted using Eureka [16], the unified access point to bibliographic resources of the University of Turin, employing the following search terms: kw: (dasatinib chylothorax) OR ti: (dasatinib chylothorax). Manuscripts focusing on case reports or case series of dasatinib-induced chylothorax published up to February 13, 2026, were included. No language or date restrictions were applied. Literature comprising systematic reviews was excluded to avoid duplication, as well as abstracts or pharmacovigilance analyses reporting incomplete data. Additionally, case reports in which thoracentesis was performed solely for diagnostic purposes and not as a therapeutic intervention were excluded from the analysis.

To organize all eligible literature, a table was created, including the following information for each study: Authors, Diagnosis, Age, Gender, Prior Therapy to Dasatinib, Daily Dose of Dasatinib, Time from dasatinib initiation to the first manifestations of chylothorax, Triglycerides (mg/dL), Clinical features, Intervention and Patient outcomes.

For the pharmacovigilance analysis, we utilized the FDA Adverse Event Reporting System (FAERS) Public Dashboard database [17]. The search was conducted using the “Search by Reaction Term” function, selecting “Chylothorax,” and subsequently choosing the drug “Dasatinib” in the interface labeled “Cases by Generic Name.” The analysis included all adverse drug reactions (ADRs) reported in the Dashboard up to September 30, 2024. The adverse reactions are categorized according to the system organ class (SOC) and described using the Preferred Term (PT) in accordance with the Medical Dictionary for Regulatory Activities (MedDRA). Given the spontaneous nature of FAERS data and the potential for duplicate reports, a rigorous deduplication process was implemented. Duplicate entries were identified and excluded based on a set of specific demographic and clinical criteria, including age, sex, country of origin, date of the event, suspected drug, type of adverse reaction, and therapeutic indication. Microsoft Excel was used to support this process.

We analyzed the following variables:

- Year of receipt of the adverse reaction report;
- Seriousness;
- Sex;
- Age group;
- Outcome.

Regarding reported outcomes, multiple outcomes could be associated with a single case. The adverse drug reactions reported in the FAERS system were analyzed, as it represents the largest global database for adverse event reporting [18]. The analysis was conducted in accordance with the STROBE (Strengthening the Reporting of Observational Studies in Epidemiology) guidelines to ensure transparency and rigor in the evaluation of pharmacovigilance data.

Since all adverse events were processed from sources with anonymized data, and the study does not include any information that could render patients identifiable, approval from the Ethics Review Board was not considered necessary.

It is important to note that FAERS is a spontaneous reporting system primarily designed for signal detection. Therefore, the data derived from this analysis are intended solely for hypothesis generation and cannot be used to establish causal relationships between dasatinib and chylothorax.

## Results

### Cases Identified from the Literature

**Fig. 1:**
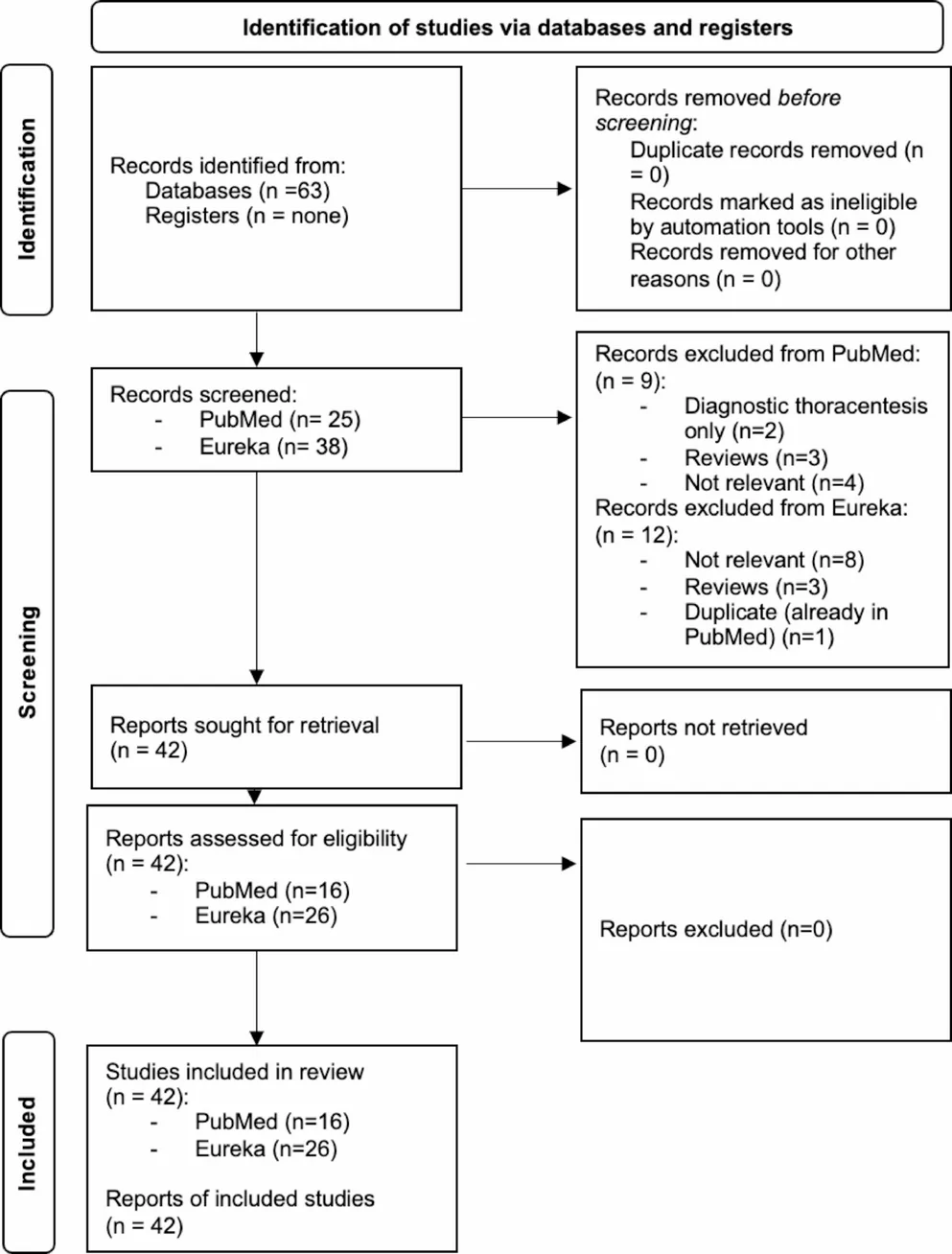
PRISMA flow diagram summarizing the study selection process

A total of 63 articles were identified through the PubMed/Medline [1–14, 19–27] and Eureka search [28–53]. After excluding studies that did not meet the search criteria, a total of 42 studies were reviewed (Fig. 1). The majority of the selected publications were case reports, primarily describing patients diagnosed with CML, except for three cases involving Philadelphia chromosome-positive (Ph +) ALL (Table 1). Across the reviewed literature, a total of 44 patients with dasatinib-associated chylothorax were reported.

**Table 1:** Summary of reported cases of dasatinib-associated chylothorax in patients with CML or Ph + ALL

Authors	Diagnosis	Age	Gender	Prior therapy to dasatinib	Daily dose of dasatinib	Time from dasatinib initiation to the first manifestations of chylothorax	Triglycerides (mg/dL)	Clinical features	Intervention	Patient outcomes
Huang et al. [19]	CML	40	F	Imatinib	100 mg	40 months	Right lung: 263, Left lung: 536	Dyspnea and aggressive cough	Steroids, diuretics, discontinuation of dasatinib, switch to nilotinib	The patient maintains her CMR status
Ferreiro et al. [6]	Ph + ALL	71	F	Imatinib	140 mg	2 months	Right lung: 625, Left lung: 378	NA	Dose reduction, corticosteroids, diuretics and thoracentesis	The response of the patient was a decrease in the PE
Baloch et al. [20]	CML	69	M	Imatinib, nilotinib	100 mg	10 months	405	Dyspena, diminished breath sounds and increased egophony on his right side	Thoracentesis, dose reduction, then discontinuation of dasatinib and switch to bosutinib	Resolution
Al-Abcha et al. [21]	CML	63	F	None	100 mg	48 months	700	Increasing shortness of breath	Prednisone, doxycycline, ceftriaxone and furosemide, thoracentesis, dose reduction, then discontinuation of dasatinib and switch to nilotinib	Resolution
Sasaki et al. [4]	CML	73	F	None	70 mg	12 months	273	Reduction in right breath sounds	Furosemide, thoracentesis, goreisan, discontinuation of dasatinib and switch to imatinib	Improved clinical profile
Chen et al. [22]	CML	71	M	Imatinib	100 mg	6 months	227	Progressive dyspnea	Thoracentesis, discontinuation of dasatinib, switch to another medication	Resolution
Hickman et al. [23]	CML	5	F	Imatinib	150 mg/m^2^/day	10 months	603	NA	Thoracentesis, discontinuation of dasatinib	Resolution
Hsu et al. [24]	CML	51	M	Imatinib	100 mg	50 months	135	Dyspnea and non-productive cough	Thoracentesis, discontinuation of dasatinib, switch to nilotinib	Resolution
Makimoto et al. [14]	CML	43	M	Imatinib, nilotinib	100 mg	123 months	1374	Dyspnea and hypoxic	Thoracentesis, discontinuation of dasatinib, switch to bosutinib	Resolution
Ng et al. [25]	CML	63	M	None	Unknown	45 months	620	NA	Thoracentesis, discontinuation of dasatinib and switch to imatinib	Resolution
Paul et al. [3]	Ph + ALL	51	M	None	140 mg	20 months	Right lung: 1346, Left lung: 1033	Fatigue, exertional shortness of breath and dizziness	Thoracentesis, prednisolone with appropriate diuresis, switching to imatinib	Resolution
McGonegal et al. [26]	CML	79	F	NA	NA	NA	442	Acute dyspnea and generalized chest pressure	Thoracentesis, discontinuation of dasatinib and switch to bosutinib	Six months later, the patient remained stable with no recurrence of chylothorax
García Martínez et al. [9]	CML	53	M	NA	NA	14 years	583	Fever, dyspnea, and abdominal pain	Thoracentesis, ocreotide, discontinuation of dasatinib	Improved clinical profile
Alqattan et al.[1]	CML	18	F	Imatinib	100 mg	63 months	584	NA	Thoracentesis, steroids, discontinuation of dasatinib and switch to nilotinib	Resolution
Liu et al.[27]	CML	11	M	Imatinib	100 mg	38 months	NA	Dyspnea characterized by decreased breath sounds on both lungs	Thoracentesis, diuretics, steroids, octreotide, fasting, discontinuation of dasatinib and switch to nilotinib	Resolution
Bradt et al.[5]	CML	67	M	None	100 mg	24 months	969	Exertional dyspnoea	Repeated thoracentesis, pleurodesis, discontinuation of dasatinib and switch to bosutinib	Recurring dasatinib-induced chylothorax even after discontinuation of this therapy
Anwar et al. [28]	CML	71	F	Imatinib	100 mg	NA	113	Mild hypoxia to 95%, decreased breath sounds bilaterally, normal thyroid, renal, and liver function	Switch to dasatinib	Slow improvement of effusions
Bogart et al. [29]	CML	84	M	NA	NA	40 months	507	Cough dyspnea	Steroids, diuretics, discontinuation of dasatinib	Resolution
Castellanos et al. [30]	CML	50	M	NA	NA	48 months	644,72	Dyspnea and non-productive cough	Thoracentesis, reduction of dasatinib	Improved clinical profile
Chua et al. [31]	CML	44	F	NA	100 mg	48 months	NA	Dyspnea	Thoracentesis, discontinuation of dasatinib	Resolution
Dalbah et al. [32]	CML	36	F	Imatinib	100 mg	4 months	1008	NA	Thoracentesis, diuretics, steroids, discontinuation of dasatinib	The patient's shortness of breath resolved and the Chest X-ray was unremarkable for pleural effusions
Espinosa et al. [33]	CML	50	M	NA	NA	48 months	425	Shortness of breath without associated symptoms	Discontinuation of dasatinib	Resolution
Grover et al. [34]	CML	65	M	NA	NA	NA	500	The patient was asymptomatic	Steroids	Patient’s pleural effusion stabilized on steroids
Haddad et al. [35]	CML	52	M	Imatinib	100 mg	60 months	485	Shortness of breath over the past 4 months	Steroids, diuretics, discontinuation of dasatinib	Improved clinical profile
Jasti et al. [36]	CML	76	M	NA	NA	NA	455	NA	Discontinuation of dasatinib, octreotide	Patient achieved chyle output reduction with eventual talc pleurodesis
Kaufman et al. [37]	CML	44	F	None	100 mg	72 months	Right lung: 317, Left lung: 656	NA	Steroids, diuretics, talc pleurodesis and bilateral tunneled IPC placement	Resolution
Kho et al. [38]	CML	53	M	None	100 mg	84 months	150,57	The patient was asymptomatic	Discontinuation of dasatinib, switch to nilotinib	Resolution
Korotun et al. [39]	CML	44	M	NA	NA	NA	610	Dyspnea for three days	Thoracentesis, discontinuation of dasatinib	Improved clinical profile
Mortada et al. [40]	CML	45	M	NA	NA	NA	177,14	Dry cough, worsening shortness of breath and left-sided chest pain	Thoracentesis	Resolution
Molina et al. [41]	CML	63	F	NA	100 mg	12 months	334	Dyspnea on exertion	Steroids, discontinuation of dasatinib, switch to imatinib	PE resolved with no sequelae observed on chest CT a month later
Natraj et al. [42]	CML	35	M	Imatinib	140 mg	11 months	1280	Dyspnea and non-productive cough	Discontinuation of dasatinib	Resolution
Oudart et al. [43]	CML	90	M	None	NA	120 months	122	Dyspnea	Discontinuation of dasatinib, switch to hydroxycabamide	Resolution
Trivedi et al. [44]	CML	62	M	NA	NA	48 months	603	Dyspnea	Thoracentesis, steroids	Improved clinical profile
Tsiouprou et al. [45]	CML	51	M	NA	NA	NA	1232	Dyspnea and non-productive cough	Discontinuation of dasatinib, switch to nilotinib	Resolution
Yang et al. [46]	CML	NA	NA	None	100 mg	8,19,24 months	NA	Dyspnea	Reduction of dasatinib	Resolution
Zengin et al. [47]	CML	21	F	Imatinib, hydroxyurea	100 mg	44 months	1133	The patient was asymptomatic	Steroids, diuretics, discontinuation of dasatinib, switch to nilotinib	Respiratory function tests are still impaired
Nikita et al. [48]	CML	79	M	None	NA	60 months	222	Acute hypoxic respiratory failure	Thoracentesis, diuretics, discontinuation of dasatinib	Improved clinical profile
Zaid et al. [49]	CML	60	M	None	NA	96 months	372	Left-sided chest pain on inspiration, dizziness, and lightheadedness	Thoracentesis, discontinuation of dasatinib, VATS	Resolution
Brodie et al. [50]	CML	45	F	None	100 mg	36 months	NA	bilateral pleural effusions and chronic hypoxic respiratory failure	Reduction of dasatinib, tadalafil, macitentan, orenitram, first switch to bosutinib and then asciminib	Improved clinical profile
Thaker et al. [51]	CML	40	M	None	100 mg	6 months	558	Dyspnea	Discontinuation of dasatinib, switch to imatinib	Resolution
Pai et al. [52]	CML	43	M	None	100 mg	72 months	738	Dyspnea and an episode of near-syncope	Steroids, diuretics, sildenafil, discontinuation of dasatinib, switch to nilotinib	Resolution
Wee et al. [53]	Ph + ALL	12	M	None	90 mg	18 months	NA	Fever, cough, and rhinorrhea lasting 3 days	Steroids, diuretics	Resolution

CML: Chronic myeloid leukemia; Ph + ALL: Philadelphia chromosome-positive acute lymphoblastic leukemia; M: Male; F: Female; NA: Not available; mg: milligrams

Patient ages ranged from 5 to 90 years, with two pediatric cases identified. Gender distribution was relatively balanced, comprising 27 males and 14 females. Many of the patients had received prior treatments before starting dasatinib, with imatinib as the main therapy, followed by nilotinib and bosutinib. Some patients, however, had not received any prior treatment before beginning dasatinib therapy.

The daily dose of dasatinib generally ranged from 50 to 140 mg per day. Treatment duration prior to the onset of chylothorax ranged from 2 months to 10 years. Reported serum triglyceride levels were often elevated, ranging from 110 mg/dL to 1374 mg/dL. Management strategies primarily included dasatinib discontinuation and therapeutic thoracentesis (pleural fluid drainage). In many cases, patients were transitioned to alternative tyrosine kinase inhibitors such as nilotinib, bosutinib, or imatinib.

### Pharmacovigilance Analysis from FAERS

The pharmacovigilance analysis included data reported in the FAERS from 2007 through September 30, 2024. A total of 594 reports related to “chylothorax” were identified, of which 124 (20.88%) listed dasatinib as the suspect drug. Dasatinib was the most frequently reported drug in relation to this adverse event on FAERS, a finding that reflects reporting patterns rather than confirmed causality.

**Fig. 2:**
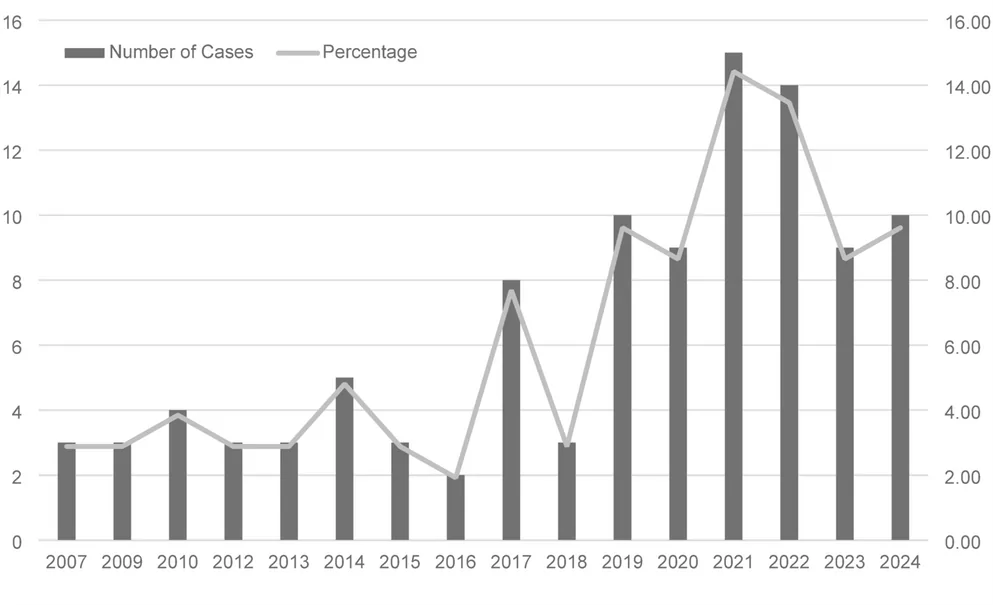
Trend of dasatinib report submissions for chylothorax over the years. Source: FDA Adverse Event Reporting System

All 124 cases involving dasatinib were classified as serious, including one fatal case. Following a deduplication process, the final number of unique cases was determined to be 104, forming the definitive dataset for analysis. The annual number of reports increased over time, with a notable rise between 2017 and 2023 and peaks observed in 2021 and 2022 (Fig. 2).

Demographic data for the 104 dasatinib-associated chylothorax reports are summarized in Table 2. Of the cases where gender was specified, 58 (55.77%) involved male patients and 37 (35.58%) involved female patients; gender was unspecified in 9 cases (8.65%).

**Table 2:** Distribution of reported cases of dasatinib-related chylothorax by gender, age group, reporter type, and outcome. The table summarizes the percentage of cases across each category. Source: FDA Adverse Event Reporting System

	Number of cases	%
*Gender*		
Female	37	35.58
Male	58	55.77
Not specified	9	8.65
Totals	104	100
*Age group*		
3–11 years	4	3.85
12–17 years	3	2.88
18–64 years	51	49.04
65–85 years	34	32.69
More than 85 years	1	0.96
Not specified	11	10.58
Totals	104	100
*Reporter type*		
Healthcare professional	95	91.35
Consumer	9	8.65
Totals	104	100
*Outcome*		
Other outcomes	98	62.42
Hospitalized	53	33.76
Disabled	3	1.91
Life threatening	2	1.27
Died	1	0.64
Totals	157	100

In terms of age, the most affected group was between 18 and 64 years, with 51 cases (49.04%), followed by the 65–85 years age group with 34 cases (32.69%). Pediatric and geriatric age groups showed much lower numbers: 4 cases between 3 and 11 years, 3 cases between 12 and 17 years, and only one case over 85 years.

Regarding the type of reporters, the healthcare professional category clearly dominated with 95 reports (91.35%), while consumers contributed only 9 reports (8.65%). This highlights the importance of professional medical surveillance in the collection and recording of adverse events.

Regarding reported outcomes, multiple outcomes could be associated with a single case. The "Other Outcomes" category was the most prevalent, with 98 cases (62.42%). Hospitalizations represented a noteworthy percentage, with 53 cases (33.76%). Severe outcomes were relatively rare: 3 cases of “Disabled” (1.91%), 2 cases classified as “Life-threatening” (1.27%), and a single “Died” case (0.64%).

## Discussion

Chylothorax is a rare but documented adverse event reported in patients treated with dasatinib. Although primarily reported through case studies and pharmacovigilance data, it represents a clinically relevant complication due to its potential impact on patient management and quality of life. This work combines a scoping review of the available literature with a pharmacovigilance analysis based on data from the FAERS. The pharmacovigilance findings presented in this study must be interpreted within the intrinsic limitations of spontaneous reporting systems. FAERS data do not allow for the assessment of incidence, risk estimation, or causal inference, as reports are subject to under-reporting, reporting bias, missing information, and lack of an exposed population denominator. Consequently, the observed signals should be considered exploratory and hypothesis-generating rather than confirmatory.

A growing number of reports describing chylothorax in patients treated with dasatinib have been submitted to the FAERS database, particularly between 2017 and 2023, with a peak in 2021 and 2022. This trend reflects greater awareness among clinicians and improved recognition of this specific complication [54]. However, it is also possible that the number of reports correlates with a broader use of dasatinib over time. Second-generation tyrosine kinase inhibitors (TKIs), such as dasatinib and nilotinib, are often preferred over imatinib due to their higher rates of early molecular response, making them first-line choices in many treatment protocols [55]. In parallel, pharmacovigilance efforts have intensified in recent years. Enhanced training for healthcare professionals, the deployment of specialized monitoring personnel within hospitals, and streamlined reporting systems have all contributed to improved adverse event detection and documentation. Additionally, external events – such as the COVID-19 pandemic and large-scale vaccination campaigns – have influenced reporting dynamics [56].

Regarding patient demographics, most reported cases involved adults aged 18–64 years, followed by those aged 65–85. Reports were infrequent in pediatric (3–17 years) and elderly (> 85 years) populations. This distribution likely reflects real-world prescribing patterns. Dasatinib is more frequently administered in adult patients with CML or Ph + ALL, while its use in elderly patients may be limited by tolerability concerns and comorbidities, often requiring dose adjustments [57]. In pediatric patients, concerns related to the potential negative impact of TKIs on growth and final height may also influence prescribing decisions [57, 58].

A slight male predominance was observed among the case reports (63.36%) and also on the real-world FAERS database (54.8%). While this may be attributed to differences in drug exposure, a biological sex-related predisposition cannot be excluded. Interestingly, literature suggests that females are generally more susceptible to adverse drug reactions [59], making this male predominance potentially noteworthy and worthy of further investigation.

The latency period between the initiation of dasatinib therapy and the onset of chylothorax varied widely across the literature, from as early as 2 months to as long as 10 years [2, 9, 10]. Although many cases occurred within the initial years of therapy, the presence of late-onset cases suggests that both acute and chronic pathophysiological mechanisms may be involved. These may include progressive vascular or lymphatic injury or delayed immunological alterations that evolve over time [33]. This variability in onset complicates risk stratification and highlights the importance of long-term monitoring for patients on dasatinib therapy.

In the reviewed case reports, serum triglyceride levels in patients with dasatinib-associated chylothorax ranged from 113 mg/dL to 1374 mg/dL. These values are consistent with the biochemical profile of chyle, which is typically rich in triglycerides, chylomicrons, cholesterol, and fat-soluble vitamins [60–62]. In clinical practice, pleural fluid triglyceride levels above 110 mg/dL are generally considered diagnostic for chylothorax, particularly when pleural cholesterol remains below 200 mg/dL. However, the presence of chylomicrons – detectable via lipoprotein electrophoresis – remains the diagnostic gold standard [60, 61]. Intermediate triglyceride values (55–110 mg/dL) may occur in both chylothorax and pseudochylothorax, necessitating further biochemical or lipoprotein analysis to confirm the diagnosis. Moreover, low triglyceride levels can occasionally be observed in true chylothorax, particularly in patients who are fasting or malnourished, highlighting the importance of clinical context when interpreting biochemical data [60].

Discontinuation of dasatinib was the most common therapeutic intervention reported both in case reports from the literature review and in FAERS cases, often accompanied by thoracentesis. Switching to another tyrosine kinase inhibitor (nilotinib or bosutinib) was feasible in several cases, with no recurrence of chylothorax reported. According to FAERS data, all dasatinib-related chylothorax cases were classified as serious adverse events, yet severe outcomes were rare. Only 1 case was reported as fatal, while 3 were associated with disability and 2 were classified as life-threatening. Hospitalizations were reported in 62 out of 185 cases (33.51%), indicating that the complication often requires inpatient care, but generally does not lead to irreversible consequences. The majority of reports fell under the “other outcomes” category, which likely includes clinical improvement following intervention or drug discontinuation.

To the best of our knowledge, this is the most comprehensive review of case reports concerning dasatinib-induced chylothorax published to date. The combined approach, including both a scoping review and pharmacovigilance analysis, strengthens the robustness of the findings by capturing both detailed clinical data from case reports and broader patterns from real-world reporting systems. In fact, while Liu et al. [63] focused exclusively on previously published case reports, our review adopts a broader methodological approach. In addition to systematically analyzing eligible case reports identified through database searches, we also integrated pharmacovigilance data extracted from the FAERS database. This allowed us to complement the evidence derived from individual case descriptions with real-world post-marketing safety data. Furthermore, our literature search was updated through February 2026, resulting in the inclusion of a larger number of cases compared with the aforementioned study. The combination of published case-based evidence and pharmacovigilance data provides a more comprehensive assessment of the clinical presentation, timing of onset, management strategies, and outcomes of dasatinib-induced chylothorax, thereby strengthening the overall robustness and clinical relevance of our findings.

This study has several limitations. First, the analysis of FAERS data is inherently constrained by the spontaneous and voluntary nature of adverse event reporting, which can lead to under-reporting, duplicate entries, and incomplete or low-quality data. Additionally, the absence of a standardized denominator (i.e., the total number of patients exposed to dasatinib) limits our ability to estimate the true incidence of chylothorax. Another key limitation is the lack of prescription data stratified by year, which makes it difficult to verify whether the observed increase in reported cases is attributable to increased dasatinib use or to improved pharmacovigilance awareness. A more detailed analysis of annual prescribing trends could help clarify this point. Furthermore, the demographic distribution of reported cases – particularly the low number of pediatric and elderly patients – raises the question of whether this pattern reflects true biological differences in susceptibility or simply mirrors real-world prescribing practices. Access to age-specific prescription data would help address this uncertainty. Another limitation of this study is the absence of prospective protocol registration. This scoping review was conducted retrospectively, and protocol registration was therefore not feasible. In addition, commonly used platforms such as PROSPERO do not consistently accept scoping reviews. Nevertheless, the methodology was defined a priori and conducted in accordance with PRISMA-ScR recommendations to ensure transparency and reproducibility.

As a future research direction, the hypothesis of a potential association between dasatinib and chylothorax could be further evaluated using large retrospective administrative or healthcare claims databases. Such data sources, by providing longitudinal exposure information, comparator groups, and adjustment for confounding factors, may allow for more robust pharmacoepidemiological analyses to better characterize the magnitude and temporal relationship of this potential risk.

## Conclusions

Chylothorax is a rare but serious adverse event reported in association with dasatinib therapy. Although relatively infrequent, its clinical impact warrants attention due to the need for hospitalization and potential disruption of ongoing treatment. The findings from case reports and pharmacovigilance data emphasize the importance of long-term monitoring, as the complication can occur even after years of treatment.

Although elevated triglyceride levels are frequently reported in dasatinib-associated cases, it remains unclear whether these alterations are a predisposing factor or a consequence of lymphatic disruption. To date, no studies have explored whether patients with baseline hypertriglyceridemia are at greater risk of developing chylothorax during dasatinib therapy. Given the central role of the thoracic duct in transporting dietary lipids, future studies could assess whether triglyceride-lowering intervention, either pharmacologic or dietary, might reduce this risk. This is particularly relevant since medium-chain triglycerides (MCTs), which bypass the lymphatic system, are already employed in conservative management of chylothorax [60–62].

In addition to dietary modification, corticosteroid therapy has been used successfully in select cases, especially when an immunologic mechanism is suspected [60]. While evidence remains limited, such adjunctive treatments may be considered on a case-by-case basis when standard measures fail or are contraindicated.

However, findings derived from spontaneous reporting systems should be interpreted cautiously and considered hypothesis-generating rather than confirmatory.

Overall, increasing awareness among clinicians, prompt recognition of pleural effusion symptoms, and a multidisciplinary approach to diagnosis and management are essential to improving outcomes in patients treated with dasatinib.

## Data Availability

The data that support the findings of this study are publicly available.
